# TGF-β1 conjugated chitosan collagen hydrogels induce chondrogenic differentiation of human synovium-derived stem cells

**DOI:** 10.1186/1754-1611-9-1

**Published:** 2015-01-14

**Authors:** Jinku Kim, Brian Lin, Soyon Kim, Bogyu Choi, Denis Evseenko, Min Lee

**Affiliations:** Department of Bio and Chemical Engineering, Hongik University, Sejong, 339-701 South Korea; Division of Advanced Prosthodontics, University of California, Los Angeles, CA 90095 USA; Department of Bioengineering, University of California, Los Angeles, CA 90095 USA; Department of Orthopaedic Surgery, University of California, Los Angeles, CA 90095 USA

**Keywords:** Chitosan hydrogels, Type II collagen, Transforming growth factor, Synovium-derived stem cells, Chondrogenic differentiation

## Abstract

**Background:**

Unlike bone tissue, articular cartilage regeneration has not been very successful and has many challenges ahead. We have previously developed injectable hydrogels using photopolymerizable chitosan (MeGC) that supported growth of chondrocytes. In this study, we demonstrate a biofunctional hydrogel for specific use in cartilage regeneration by conjugating transforming growth factor-β1 (TGF-β1), a well-documented chondrogenic factor, to MeGC hydrogels impregnating type II collagen (Col II), one of the major cartilaginous extracellular matrix (ECM) components.

**Results:**

TGF-β1 was delivered from MeGC hydrogels in a controlled manner with reduced burst release by chemically conjugating the protein to MeGC. The hydrogel system did not compromise viability of encapsulated human synovium-derived mesenchymal stem cells (hSMSCs). Col II impregnation and TGF-β1 delivery significantly enhanced cellular aggregation and deposition of cartilaginous ECM by the encapsulated cells, compared with pure MeGC hydrogels.

**Conclusions:**

This study demonstrates successful engineering of a biofunctional hydrogel with a specific microenvironment tailored to promote chondrogenesis. This hydrogel system can provide promising efficacious therapeutics in the treatment of cartilage defects.

**Electronic supplementary material:**

The online version of this article (doi:10.1186/1754-1611-9-1) contains supplementary material, which is available to authorized users.

## Background

More than 70 million adults in the United States suffer from articular cartilage injuries caused mainly by arthritis. An estimated economic burden to arthritis-related disability would be $100 billion by 2020 as elderly population continues to grow [1]. Although autologous chondrocyte implantation (ACI) is clinically available for articular cartilage injuries, successful regeneration of damaged articular cartilage remains a great challenge due to the limitations (e.g. multiple surgical procedures, in vitro cell expansion) associated with ACI [2–5]. Consequently, tissue engineering approaches have been utilized for articular cartilage regeneration [6, 7]. However, unlike bone, cartilage regeneration using tissue engineering strategies has not been successful due to unfavorable selection of appropriate cell sources, scaffolds and/or biomolecules to precisely restore structure and function of the elegantly organized tissue [8, 9].

Recently, in an effort to remove harsh conditions and chemicals used in scaffold fabrication, our laboratory have developed the chitosan-based hydrogel system by utilizing mild visible blue light (VBL) and riboflavin (vitamin B2) as a light source and an initiator, respectively, instead of popular choices of UV light and harmful photoinitiators [10, 11]. As a result, the delivery system bio-degraded to match growth rates of cartilage regeneration and removed harsh chemicals while maintaining chondrocyte morphology and phenotype, which will be crucial for cartilage regeneration during chondrogenesis of mesenchymal stem cells (MSCs) [12]. Yet, systematic tissue engineering approaches is still required for successful cartilage repair.

Among numerous biomolecules for cartilage repair, transforming growth factor-β1 (TGF-β1) has been explored to enhance chondrogenic differentiation of cartilage forming cells as well as biomechanical properties of neocartilage [13, 14]. However, therapeutic efficacy of TGF-β1 is affected by delivery kinetics due to its intrinsic protein instability and rapid enzymatic degradation in vivo requiring a high therapeutic dose. The high dose requirement can cause adverse side effects such as fibrotic disorders and unwanted osteophyte formation in the synovium [15, 16]. Sulfated polysaccharides such as heparin have been shown to form stable complexations with TGF-β1, which maintain the biological activity and can sustain the protein release [17–20]. Recent studies have used this affinity binding of heparin for the controlled delivery of TGF-β1 with prolonged bioactivity [21–23]. However, such a physical adsorption-based delivery approach requires a high amount of growth factors and is easily affected by a local microenvironments causing burst release of the loaded protein. Chemical conjugation of growth factors into scaffolds showed a more prolonged release and significantly reduced initial burse of the proteins compared to physically absorbed growth factors [24, 25].

In addition, type II collagen (Col II) is known to be the most abundant protein in the cartilage tissue and to promote chondrogenic differentiation of MSCs [26, 27]. Previous studies demonstrated that TGF-β1-mediated chondrogenesis was enhanced significantly in the presence of Col II [28–30]. Chondrocytes bind to Col II through integrins leading to the formation of signaling complex that induces chondrogenesis. TGF-β1 interacts with a receptor complex and transduces its signals through phosphorylation of the cytoplasmic signaling molecules (Smads). It has been demonstrated that combination of TGF-β1 and Col II treatment resulted in a synergistic increase in Smad 2 and 3 phosphorylation compared with the individual stimulation with TGF-β1 or Col II alone, indicating the signaling cross-talk between Col II-activated integrin pathway and the TGF-β1 Smad pathway [28–30].

Here, we introduce the sophisticated cartilage tissue engineering system supplemented with TGF-β1 bioconjugation and Col II impregnation into the methacrylated chitosan (MeGC) hydrogels to promote chondrogenic differentiation of encapsulated MSCs derived from human synovium (hSMSCs). We hypothesized that the TGF-β1 conjugation and Col II impregnation into the chitosan hydrogels will additively enhance chondrogenic differentiation of encapsulated hSMSCs. To test this hypothesis, we encapsulated hSMSCs into the MeGC hydrogels functionalized with Col II and TGF-β1 and determined the ability of the hydrogel systems to promote chondrogenesis using a series of in vitro assays at different time courses up to 21 days.

## Results

### Hydrogel fabrication and characterization

**Figure 1:**
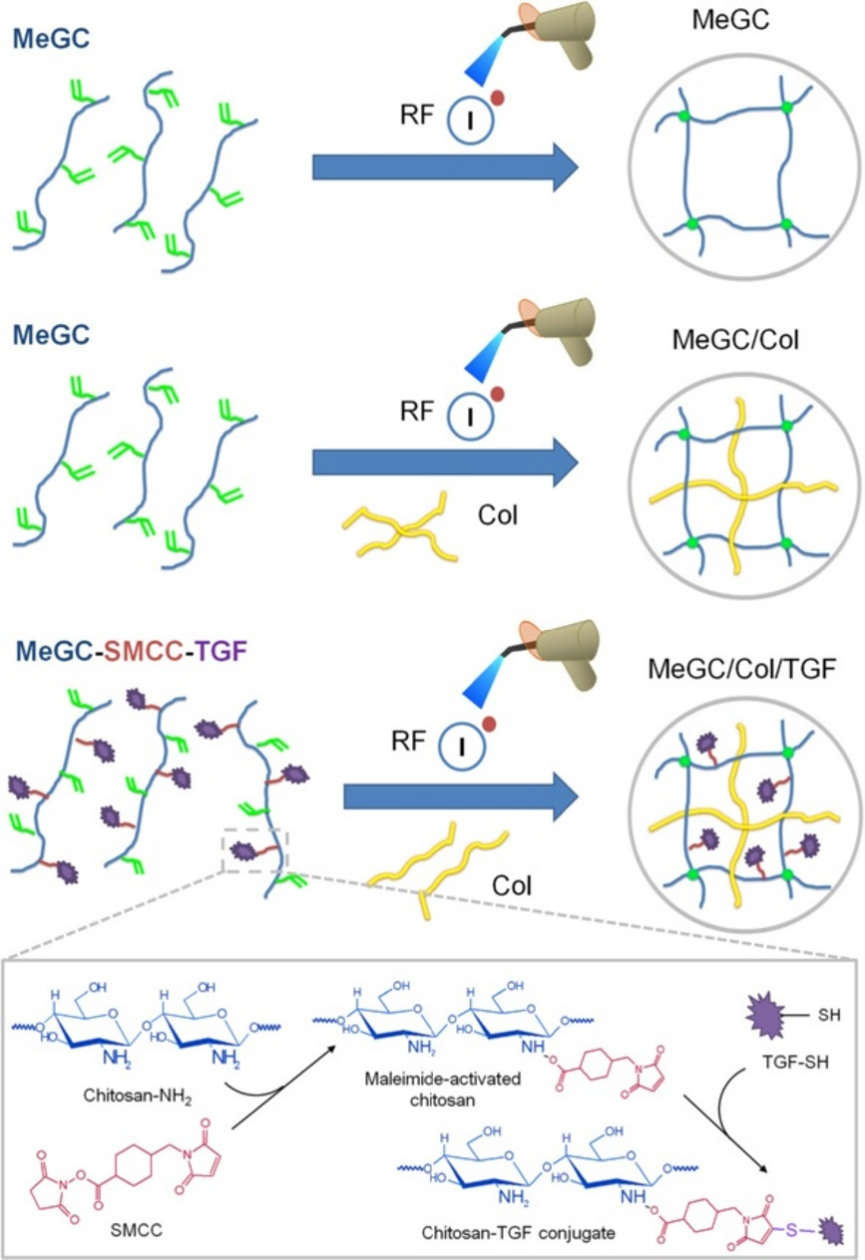
**Schematic diagram of functionalized MeGC hydrogels.** MeGC hydrogels were formed by irradiation of VBL in the presence of RF as a photoinitiator. MeGC/Col hydrogels were prepared by mixing MeGC solution with Col II followed by photopolymerization. TGF-β1 was bioconjugated into MeGC via SMCC moiety.

**Figure 2:**
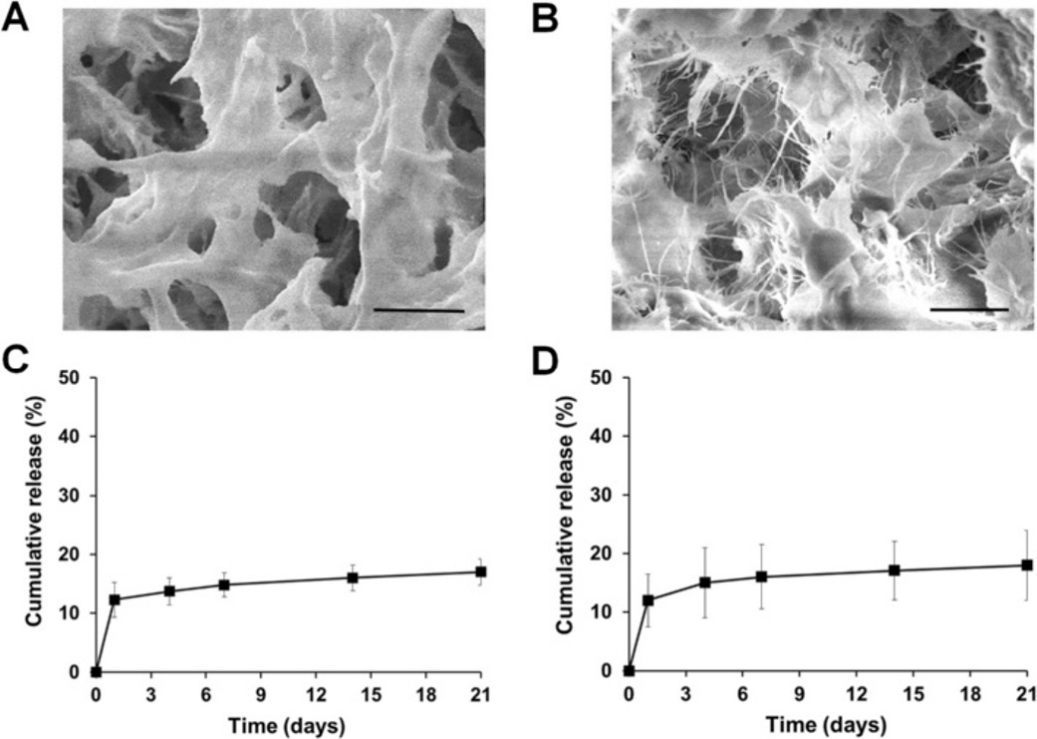
**Hydrogel characteristics and release kinetics.** SEM images of MeGC **(A)** and MeGC/Col **(B)** hydrogels. Scale bar = 10 μm. Release profiles of TGF-β1 from MeGC/TGF **(C)** and MeGC/Col/TGF **(D)** hydrogels incubated in culture media with serum.

Chitosan (MeGC) and Col II-impregnated chitosan hydrogels (MeGC/Col) were prepared via free radical polymerization under VBL in the presence of a RF initiator (Figure 1). TGF-β1 was chemically conjugated into MeGC prior to hydrogel formation using a Succinimidyl-4-(N-maleimidomethyl)cyclohexane-1-carboxylate (SMCC) linker (MeGC/Col/TGF). Cross-sectional SEM images of MeGC/Col suggested that MeGC hydrogels had fibrous collagen microstructure homogeneously distributed throughout the hydrogels (Figure 2B). The conjugation of TGF-β1 onto MeGC did not alter the microstructure of the cross-linked hydrogels.

Release of conjugated TGF-β1 from MeGC hydrogels was measured in a culture medium containing serum. As expected, the release profiles from the hydrogels showed a sustained release with a reduced initial burst and approximate cumulative release of 12% up to 21 days (Figure 2C). The addition of Col II did not significantly affect the release kinetics of TGF-β1 from the hydrogel (Figure 2D). Our previous data showed that approximately 60% of initially loaded TGF-β1 by an adsorption method was rapidly released from the hydrogels at day one in a similar condition (Additional file 1: Figure S1). Chemically conjugated TGF-β1 was better immobilized on the hydrogels, thus much less amount of the protein was released from the delivery system, compared with weakly adsorbed TGF-β1. SMCC is one of the widely used crosslinking agents containing an amine-reactive N-hydroxysuccinimide (NHS) ester and a sulfhydryl-reactive maleimide group [31]. NHS esters react with the primary amino groups (-NH2) of MeGC to form stable amide bonds. Maleimide groups form covalent crosslinks with sulfhydryl (-SH) moieties on cysteine residues of TGF-β1 to form stable thioether bonds. A similar conjugation technique using SMCC was successfully employed to immobilize various growth factors, including latent TGF-β1, bone morphogenetic protein-2 (BMP-2), and vascular endothelial growth factor (VEGF) onto biomaterial surfaces, and the conjugated growth factors maintained their bioactivity [24, 32–34]. However, the conjugation process and chemistry of reaction can adversely affect the growth factor by protein denaturation and impair the biological function of the growth factor. Additional studies on protein conformational changes are needed for the future clinical translation.

### Cell growth in MeGC/Col/TGF hydrogels

**Figure 3:**
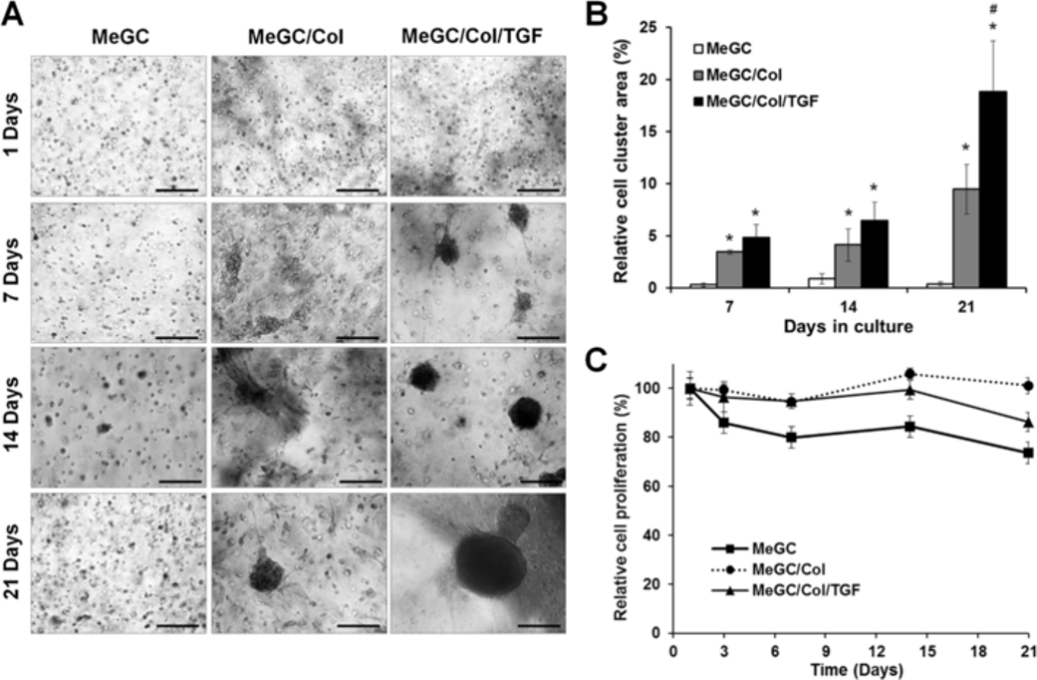
**Growth of hSMSCs cultured in hydrogels. (A)** Bright field images of hSMSCs in the hydrogels. Scale bar = 200 μm. **(B)** Percentage area of cell clusters quantified by image analysis of bright field images. (*: *p* < 0.05 compared with MeGC and #: *p* < 0.05 compared with MeGC/Col, n = 3) **(C)** Proliferation of hSMSCs in hydrogels measured by CCK assay.

**Figure 4:**
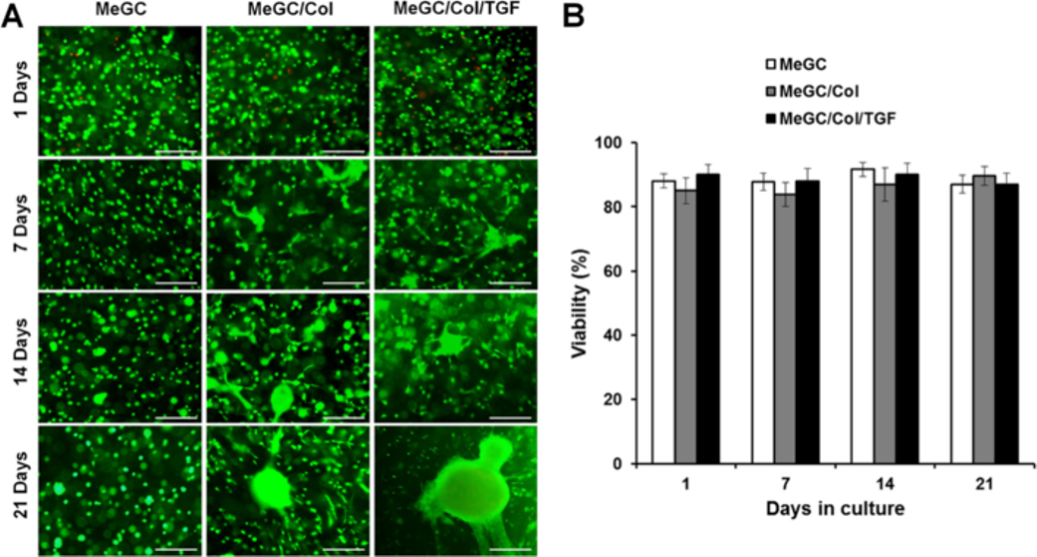
**Viability of hSMSCs cultured in hydrogels. (A)** Live/Dead staining of hSMSCs in the hydrogels and **(B)** hSMSCs viability (%) from live/dead image analysis. Scale bar = 200 μm.

The bright field microscopic images of encapsulated hSMSCs cultured in MeGC, MeGC/Col, or MeGC/Col/TGF hydrogels showed that there was little cell aggregation at day 1 regardless of the presence of Col II and/or TGF-β1 (Figure 3A). Cell aggregation was rarely observed in MeGC hydrogels over the 21-day culture period. In contrast, MeGC/Col hydrogels appeared to have a few clusters at day 7 and the area of cell clusters was increased over time. For TGF-β1 conjugated gels, much bigger cell clusters were observed after 7 days compared with other hydrogels. The image analysis data of cell cluster area in each hydrogel confirmed that there were significant differences in cell cluster area between treatment groups and pure MeGC groups at day 7, 14 and 21 days (Figure 3B). In particular, there were significant differences between MeGC/Col and MeGC/Col/TGF hydrogels at day 21, indicating that the conjugated TGF-β1 significantly promoted cell aggregations in the hydrogels. Regarding cellularity of encapsulated hSMSCs, the data revealed that MeGC/Col gels had greatest cellularity as compared to other hydrogels at days 14 and 21. MeGC/Col/TGF hydrogels had significant increase of cell proliferation, compared with the MeGC hydrogels (Figure 3C).The Live/Dead staining images showed that most cells encapsulated in the all tested hydrogels (>90%) were viable even at day 21 and there were no significant differences in cell viability among tested hydrogel systems, indicating that the use of SMCC, VBL, and RF all together had no adverse effects on cell viability (Figure 4).

### Chondrogenic differentiation of encapsulated hSMSCs

**Figure 5:**
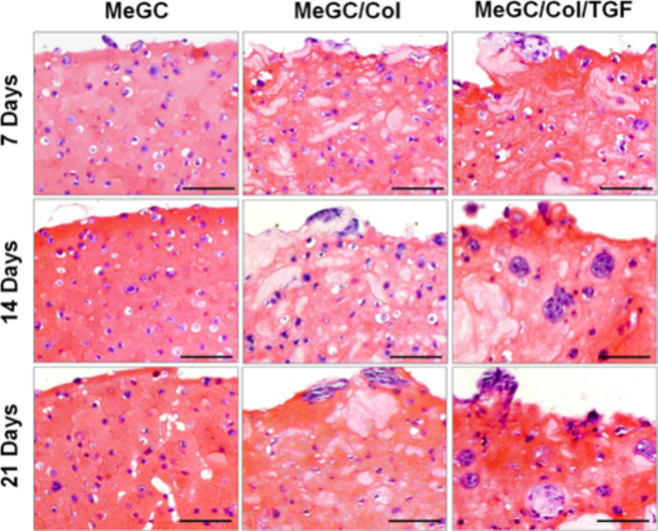
**H & E staining of hSMSCs cultured in hydrogels at days 7, 14, 21 in culture.** The encapsulated cells maintained round cell morphology throughout the culture periods. The cell aggregations were additively promoted with Col II and TGF-β1 supplementations. Scale bar = 100 μm.

**Figure 6:**
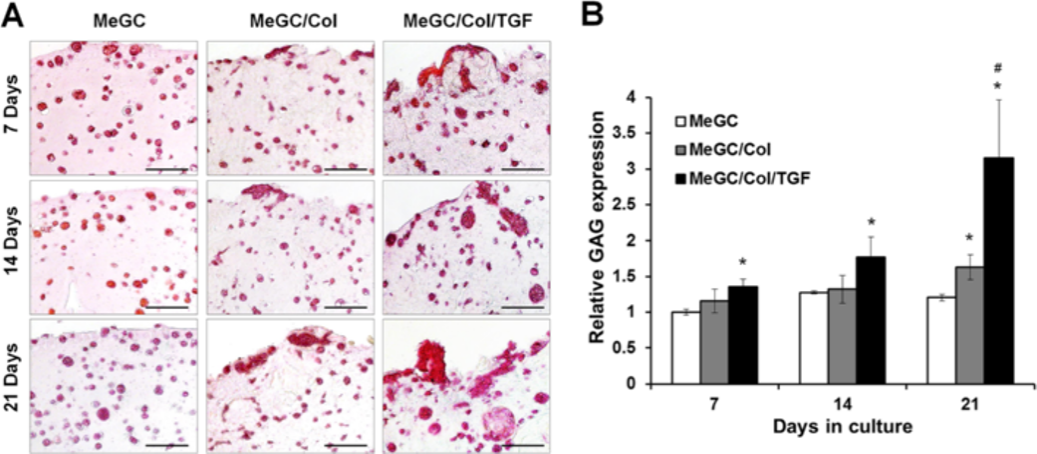
**Differentiation of hSMSCs cultured in hydrogels. (A)** Safranin-O staining of hSMSCs cultured in hydrogels and **(B)** quantification of Safranin-O staining by image analysis**.** Scale bar = 100 μm. (*: *p* < 0.05 compared with MeGC and #: *p* < 0.05 compared with MEGC/Col, n = 3).

**Figure 7:**
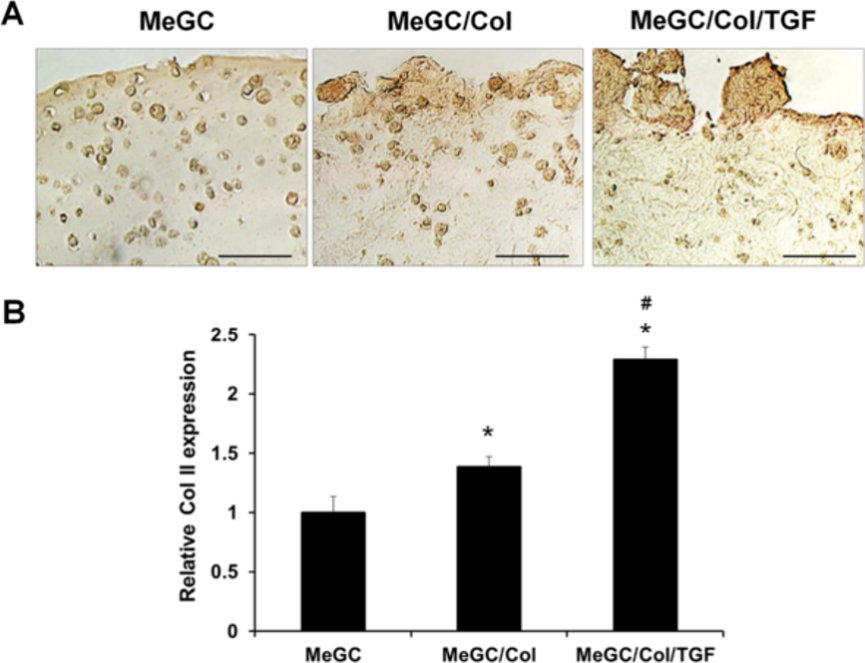
**Differentiation of hSMSCs cultured in hydrogels. (A)** Type II collagen staining of hSMSCs cultured in hydrogels at 21 days and **(B)** quantification of Col II staining by image analysis. Scale bar = 100 μm. (*: *p* < 0.05 compared with MeGC and #: *p* < 0.05 compared with MeGC/Col, n = 3).

To observe in vitro chondrogenic differentiation of encapsulated hSMSCs, histology (H&E staining and Safranin-O staining) and immunohistochemistry (Col II staining) were performed in this study.H&E staining images revealed homogeneous cell distribution and most of encapsulated cells were round shape with surrounding lacunae in all tested hydrogels at day 7, which is the characteristic of chondrogenic differentiation (Figure 5). Over the culture period up to day 21, there were no notable differences in cellular activities in MeGC hydrogels. However, cellular aggregations were observed in MeGC/Col and MeGC/Col/TGF gels with more intense cell clusters in TGF-β1-conjugated gels than MeGC/Col gels.To assess chondrogenic differentiation of encapsulated hSMSCs, safranin-O staining and Col II immunohistochemistry (IHC) were performed to detect sulfated glycosaminoglycan (sGAG) production and Col II deposition from the encapsulated cells. Increased GAG accumulation was observed over time in MeGC/Col and MeGC/Col/TGF gels, whereas pure MeGC gels did not show any differences in Safranin-O staining density over time (Figure 6A). Quantification of GAG accumulation by image analysis confirmed that there were significant differences between MeGC gels and MeGC/Col/TGF gels at all time periods and between MeGC/Col gels and MeGC/Col/TGF gels at day 21 (Figure 6B).Regarding Col II IHC at day 21, all tested groups showed positive staining for Col II (Figure 7A). In MeGC gels, Col II positive staining was observed throughout the hydrogels but limited inside lacunae, whereas the Col II staining region was extended into the area surrounding the lacunae in MeGC/Col gels. Highly intense positive Col II staining was found in MeGC/Col/TGF gels compared with MeGC/Col gels. Quantification of Col II staining verified these findings that Col II expression of encapsulated hSMSCs was significantly higher in MeGC/Col and MeGC/Col/TGF gels at day 21, compared with MeGC gels (Figure 7B).

The cellular activities of encapsulated hSMSCs in MeGC gels revealed that delivery of TGF-β1 without Col II impregnation in MeGC gels did not show any significant effects on cellular activities, compared with pure MeGC gels without TGF-β1 (Additional file 2: Figure S2).

**Figure 8:**
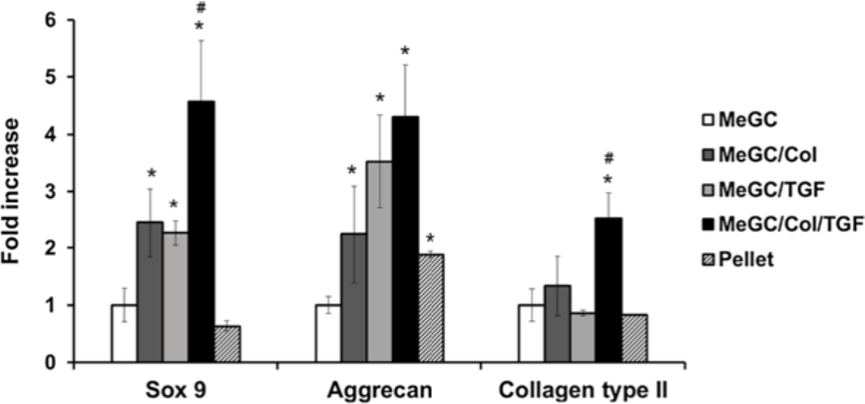
**Gene expression of** ***Sox 9*** **,** ***aggrecan*** **, and** ***Collagen type II*** **in hSMSCs cultured in hydrogels or pellets on day 14.** (*: *p* < 0.05 compared with MeGC and #: *p* < 0.05 compared with MeGC/Col or MeGC/TGF, n = 3).

Chondrogenic differentiation of hSMSCs cultured in the hydrogels was further confirmed by qRT-PCR analysis (Figure 8). hSMSCs cultured in pellets were used as a positive control, as the pellet culture system is widely used to induce chondrogenesis of MSCs [35–37]. MeGC/Col/TGF gels significantly increased the expression level of chondrogenic gene markers *Sox 9*, *aggrecan*, and *Col II* in hSMSCs, relative to MeGC gels. mRNA level of *Col II* was increased 2.5-fold in the MeGC/Col/TGF gels compared to the MeGC gels, whereas no significant change was observed in the MeGC/Col or MeGC/TGF gels.

## Discussion

The primary purpose of this study is to assess whether Col II nanofiber impregnation and TGF-β1 bioconjugation into MeGC hydrogels contribute chondrogenic differentiation of encapsulated hSMSCs in the gels. The results of the current study support the hypothesis that the Col II impregnation and TGF-β1 conjugation into the chitosan hydrogels additively promoted chondrogenic differentiation of the encapsulated hSMSCs.

Although our previous report demonstrated that MeGC-based hydrogels prepared by VBL and RF, proven to be biocompatible and to promote ECM production (e.g., GAG accumulation) of encapsulated MSCs, [10] those matrix synthesis may be minimal, thus the supplementation of exogenous growth factors or recruiting endogenous ones may be needed to facilitate chondrogenesis [38, 39]. Among soluble biomolecules to enhance cartilage formation, TGF-β1 was selected in this study due to its well-known chondrogenic potentials [14, 40]. Furthermore, Col II was also impregnated into the hydrogel network to further accelerate chondrogenic differentiation since Col II is a main component in the ECM of articular cartilage [6]. Therefore, in the present study, sophisticated functional scaffolds with a chemical moiety (i.e., SMCC) were designed to eventually match unmet needs for functional cartilage regeneration.

The Col II concentration of 0.4% was optimized in our previous study to promote chondrogenesis in MeGC hydrogels [41]. Our previous study showed that the incorporation of Col II (0.4% w/v) increased the compressive modulus of the hydrogel from 4.6 to 7.0 kPa [41]. We did not investigate higher concentrations because Col II concentrations over 0.4% were too viscous to handle and resulted in non-uniform mixing with MeGC. The final concentration of 10 μg/ml was chosen for the current studies because TGF-β1 concentrations above 10 μg/ml reduced the mechanical properties of the cross-linked hydrogel. This may be due to the reduced crosslinking density or interactions between polymer chains by the conjugated proteins. Although the mechanical properties of the hydrogels were lower than those of native cartilage, our hydrogel system was designed to mainly serve as an appropriate carrier for stem cells and growth factors, and the degrading hydrogel will be replaced by nascent cartilage tissue in vivo with sufficient mechanical support. Additional studies on the mechanical integrity of the construct in cartilage defects could further enhance the future clinical translation.

Our release data demonstrated the sustained release of TGF-β1 chemically conjugated on hydrogel via an SMCC crosslinker. The observed release may be due to hydrolysis of covalent linkages in the environment of serum-supplemented media that present various proteins, electrolytes, hormones, and enzymes. Similar observations of sustained release have been reported using BMP-2 conjugated on biomaterial surfaces via SMCC [24, 33]. It is also possible that material surfaces can affect chemical stability of the conjugates and the release pattern. It has been demonstrated that different biomaterials showed distinctive release pattern of covalently bound BMP-2 using the same crosslinker [42], indicating that the substrate material as well as the conjugation strategy can affect the release pattern. Further studies to elucidate the conjugation and release mechanisms are needed to determine appropriate linkage for the TGF-β1 conjugation. Although positively charged TGF-β1 (p*I* = 9.5) can interact electrostatically with Col II, the addition of Col II in MeGC hydrogels did not suppress the release of TGF-β1 from the hydrogels. This is possibly due to the additional proteins in serum-supplemented media that abolished the electrostatic interaction effects. Similar observations of increased release by serum proteins have been reported in our previous study using Col-coated scaffolds and cationic histone [43]. In contrast, approximately 70% of the initially loaded TGF-β1 by a non-specific adsorption method was released from the MeGC/Col hydrogels during the first week (Additional file 1: Figure S1). We observed a fewer number of cell aggregations in these hydrogels with TGF-β1 adsorption compared with the TGF-β1-conjugated hydrogels, indicating that the chemical conjugation strategy provides more favorable microenvironment to induce chondrogenesis. Similar release kinetics dependent osteogenic activity was observed in BMP-2 loaded polycaprolactone scaffolds [33]. Previous studies also revealed that sustained release of growth factors to the designated sites may further enhance cellular responses leading to desired morphogenesis of progenitor cells [44, 45].

While individual use of SMCC, visible blue light (VBL) and riboflavin (vitamin B2) has proven to be biocompatible [46, 47], it is crucial to evaluate the bioactivity of the integrated system all together. The Live/Dead staining images and proliferation data revealed that the encapsulated hSMSCs in MeGC-based hydrogels was highly viable throughout the scaffolds (>90%) despite the use of bioconjugation agent (i.e., SMCC). Furthermore, the proliferation data showed that the proliferation of hSMCSs in MeGC/Col/TGF was lower compared to the cells in MeGC/Col, suggesting that the conjugated TGF-β1 on the hydrogels may promote the encapsulated hSMSCs to undergo chondrogenic differentiation after 14 days of culture. However, hSMSCs encapsulated in MeGC/TGF hydrogel displayed a similar proliferation rate to the MeGC group and most of the seeded cells were present as individual cells without forming aggregation over 21 days, indicating that TGF-β1-mediated chondrogenesis is not sufficient without collagen interaction. Previous studies demonstrated that chondrogenic differentiation was most predominant in the cells stimulated with TGF-β1 in the presence of Col II, indicating synergistic effects of Col II binding and TGF-β1 signaling on chondrogenesis [28–30].

It is known that cell-matrix interaction plays important role in cartilage tissue engineering and previous reports suggest that cell morphology depends on scaffold type, composition and architecture [48, 49]. In cartilage development, the round cell morphology has to maintain for chondrogenic differentiation to promote cartilage-specific ECM production. However, the predominant cell morphology observed on most scaffolds except for hydrogels has been known to be elongated fibroblastic morphology, which triggers cell spreading and fibrous matrix deposition [50]. Thus, it is not surprising to use hydrogels for cartilage tissue engineering ranging from natural hydrogels such as hyaluronic acids to synthetic ones such as polyethylene (PEG)-based hydrogels [51, 52]. However, the cells encapsulated in hydrogels often lose their chondrogenic phenotype with elongated morphology, depending on hydrogel types and culture conditions [47, 49]. Therefore, it is crucial to design appropriate microenvironments to promote and maintain chondrogenic differentiation with round (or spherical) cell morphology during cell growth [53].

Cell-cell interactions are important steps in mediating an early mesenchymal condensation and chondrogenesis. The condensation events are known to be initiated by cell-cell adhesion mediated by cell adhesion molecules (N-cadherin, N-CAM) as well as cell-matrix interactions (collagen, aggrecan) [54–57]. Collagen has been shown to induce mesenchymal condensation and subsequent chondrogenesis via integrin-mediated cell adhesion by enhancing cell-matrix interactions [58–60]. Chondrogenic differentiation is further modified by the involvement of various growth factors (TGF-β, Wnt, fibroblast growth factors) that initiate intracellular signaling pathways transduced by protein kinase C and mitogen-activated protein kinases [28, 61, 62]. One of the most encouraging findings in the present study is that MeGC hydrogels supported close cell-cell contact and round cell morphology maintained throughout the culture periods with increased ECM production such as GAG and Col II expression over time. Moreover, Col II impregnation did not compromise cell morphology despite its fibrous nature. It is possible to speculate that cell morphology of the encapsulated hSMSCs maintained spherical morphology since their size is greater than that of collagen fibers [48]. Furthermore, we demonstrated that incorporation of Col II and TGF-β1 into the MeGC hydrogels at least additively increased density of cell clusters and cartilage-specific ECM production in the hydrogels. Given that the Col II initially incorporated in the hydrogel was obtained from chicken sternal cartilage and the primary antibody used for IHC has very little cross-reactivity with chicken, the observed Col II staining indicates cartilaginous matrix produced by the encapsulated cells, not the Col II initially added in the hydrogel.

There has been a controversy whether exogenous scaffolds are necessary for cartilage development as opposed to bone regeneration, where a general consensus that exogenous three dimensional (3D) scaffolds are required for the tissue development [12, 63, 64]. However, it is our point of view and others’ as well that ‘functional’ 3D scaffolds may be needed for cartilage tissue engineering to immobilize implanted cells in the defect site and to facilitate ECM deposition via cell-scaffold interactions during the tissue development [65]. Overall, MeGC hydrogels supplemented with Col II impregnation and TGF-β1 bioconjugation may be an attractive synthetic ECM for cartilage tissue regeneration.

## Conclusions

To enhance chondrogenic differentiation of encapsulated cells, functional chitosan hydrogels were developed by incorporating TGF-β1 and nanofibrous Col II into photocrosslinkable MeGC. Controlled delivery of TGF-β1 with reduced burst release was demonstrated via bioconjugation of the protein to the hydrogels. The hydrogel system did not compromise viability of encapsulated hSMSCs and the addition of Col II and TGF-β1 promoted cellular aggregation and chondrogenic differentiation of the encapsulated cells. This MeGC-based hydrogel system provides a specific microenvironment tailored to promote chondrogenesis in the treatment of cartilage defects.

## Materials and methods

### Materials

Glycol chitosan (GC: molecular weight ~500 kDa), glycidyl methacrylate, riboflavin (RF) sodium salt, and type II collagen (Col II) purified from chicken sternal cartilage were all purchased from Sigma-Aldrich (MO, USA) and used as received. Succinimidyl-4-(N-maleimidomethyl)cyclohexane-1-carboxylate (SMCC) was purchased from Pierce (Rockford, IL). Recombinant human TGF-β1 was obtained from PeproTech (Rocky Hill, NJ). Human SMSCs were provided from Dr. Denis Evseenko (UCLA Orthopedic Surgery).

### Preparation of photocrosslinkable hydrogels

Methacrylated glycol chitosan (MeGC) was prepared as previously reported [10, 47]. Briefly, glycidyl Methacrylate was added to an aqueous solution of 2% (w/v) glycol chitosan (pH 9.0) with 1.1 molar ratio of glycidyl methacrylate to the primary amine groups in chitosan then proceed to react via gentle shaking at room temperature for 36 h. The reaction mixture was then neutralized, dialyzed against water for 15 h using membrane with cutoff molecular weight of 50 kDa, and lyophilized for further studies. The degree of methacrylation to the GC was 26% as determined via ^1^H-NMR.

The composite solution of 2% w/v MeGC containing 0.4% w/v Col (MeGC/Col) was prepared by mixing stock MeGC solution (4% w/v, in PBS) with Col (1.0% w/v, in 0.05% acetic acid). Pure MeGC solution (2% w/v) was prepared by diluting the 4% w/v MeGC in PBS. The hydrogel was formed by exposing the solution to visible blue light (400–500 nm, 500–600 mW/cm^2^, Bisco Inc., Schaumburg, IL) in the presence of RF photoinitiators (6 μM).

The interior morphology of hydrogels was observed using scanning electron microscopy (SEM, Nova NanoSEM 230, FEI, Hillsboro, OR). The hydrogels were fixed with 2.5% glutaraldehyde for 2 h at room temperature and interior morphology was imaged in low vacuum mode.

### TGF-β1 conjugation

TGF-β1 was covalently conjugated to MeGC via SMCC linker. Briefly, 20 μL of SMCC (7.4 mg/mL) was added to 10 mL of MeGC (2% w/v in PBS) and incubated for 15 h at room temperature with gentle shaking. The reaction mixture was then dialyzed against water and lyophilized to receive MeGC-SMCC. To conjugate TGF-β1 to MeGC-SMCC, 10 μg of TGF-β1 in PBS was reacted with 2 mL of MeGC-SMCC aqueous solution (1% w/v). Reaction was performed for 15 h under mild shaking at 4°C and then purified with ultrafiltration tubes (MWCO 100 kDa) according to the manufacturer’s manual (Millipore). The concentrated product was recovered, lyophilized, and stored at -20°C for further study.

To examine the release kinetics of proteins, TGF-β1 tethered hydrogels (MeGC/Col/TGF) containing cells were prepared as described above and the obtained hydrogels were incubated in DMEM supplemented with 10% fetal bovine serum (FBS) at 37°C. The final concentration of TGF-β1 in hydrogels was 10 μg/mL. The incubating medium was replaced with fresh medium at the designated time interval. The amount of released TGF-β1 in the medium was measured by using an enzyme-linked immunosorbent assay (ELISA) kit (R&D systems, Minneapolis, MN). Measurements were performed in triplicate, and the amount of protein release was expressed as a percentage of the initial amount of incorporated protein.

### Culture of cells in hydrogels

Human SMSCs were expanded in DMEM with 20% FBS, 100 μg/mL streptomycin, and 100 U/mL penicillin at 37°C in a 5% CO_2_ humidified atmosphere. SMSCs (passage 3–4) were suspended in 40 μl of MeGC, MeGC/Col, and MeGC/Col/TGF-β1 solutions at a density of 10 × 10^6^ cells/mL (final concentration of TGF-β1 was 10 μg/mL). The hydrogels were cultured in chondrogenic medium consisting of DMEM with 10% FBS, ITS+ Premix supplement (BD Biosciences, Bedford, MA), 100 nM dexamethasone, 40 μg/mL L-proline, 1 mM sodium pyruvate, and 50 μg/mL L-ascorbic acid 2-phosphate (all Sigma-Aldrich) for up to 21 days and the medium was replaced twice a week.

The growth of SMSCs in the hydrogels was observed using a light microscope (Olympus IX71, Olympus, Lake Success, NY). Images of SMSCs in the hydrogels were captured from three randomly chosen fields. The cluster area was quantified from captured images using NIH-Image J software (http:/rsb.info.nih.gov/ij/). Ten to twenty clusters were counted for each hydrogel sample. Proliferation of cells was measured using Cell Counting Kit-8 (CCK-8, Dojindo, Kumamoto, Japan) according to the manufacturer’s protocol. To observe the cell viability, cell/hydrogels constructs were washed once with PBS and stained with calcein/ethidium homodimer using a LIVE/DEAD assay kit (Invitrogen, Carlsbad, CA) at 37°C for 30 min. Stained samples were observed by a fluorescent microscopy (Olympus IX71 microscope). The percentage viability was determined by calculating the number of live cells (green) normalized to the total number of cells (green and red). All the experiments were performed in triplicate (n = 3 per group).

### Histological and immunohistochemical analyses

For histological analysis, cultured hydrogels were fixed with 10% neutral buffered formalin, embedded in paraffin, then sectioned at 5 μm. The sections were deparaffinized then stained with hematoxylin and eosin (H & E) to examine cellular distribution and morphology. Safranin-O staining was performed to assess glycosaminoglycans (GAG) synthesis. Immunohistochemistry (IHC) was performed to determine Col synthesis. Briefly, sections were incubated with primary antibody against Col (anti-human Col; EMD Millipore, Billerica, MA) and antibody was detected using the SuperPicture™ polymer detection kit with DAB substrate (Invitrogen) per the manufacturers’ instructions. Images were obtained using Olympus IX71 microscope. GAG and Col production was quantified by image analysis of three randomly selected fields of each of three Safranin-O staining or Col IHC samples (n = 9/group) relatively quantified by using NIH-ImageJ software.

### RNA extraction and quantitative real-time polymerase chain reaction (qRT-PCR)

To investigate the gene expressions of SMSCs in the hydrogels, SMSCs-laden MeGC, MeGC/Col, MeGC/TGF, and MeGC/Col/TGF-β1 hydrogels were cultured in chondrogenic media for 14 days. Total RNA was extracted using Trizol reagent and RNeasy Mini Plant kit (Qiagen, Valencia, CA) as previously described [66]. Briefly, 0.5 μg of total RNA was reversely transcribed to cDNA using a cDNA transcription kit. The expressions of chondrogenic gene markers *Sox 9*, *aggrecan*, and *Col II* were measured by quantitative real-time PCR using LightCycler 480 PCR (Indianapolis, IN) with 20 μl SYBR Green reaction volume. SMSCs cultured in pellets were used as a standard of comparison. SMSC pellets were prepared as previously described [36] and cultured in chondrogenic medium supplemented with 10 ng/mL TGF-β1. The primers were designed as previously described [67]. The levels of gene expression were normalized with *GAPDH*. The amount of mRNA expression was expressed as a ratio to the MeGC hydrogel.

### Statistical analysis

Statistical analysis was performed using one way analysis of variances (ANOVA) followed by Tukey’s post hoc test. A value of *p* < 0.05 was considered statistically significant.

## Electronic supplementary material

Additional file 1: Figure S1: Supplementary data of TGF-β1 release **(A)** and SMSC growth **(B)** in MeGC/Col containing non-specifically adsorbed TGF-β1. Scale bar = 200 μm. (TIFF 10 MB)

Additional file 2: Figure S2: Supplementary data of SMSC cultured in MeGC/TGF without collagen at day 21 including **(A)** Bright field images, **(B)** live/dead staining, **(C)** H & E staining, and **(D)** Safranin-O staining. Scale bar = 100 μm. (TIFF 4 MB)
