# Supplementary figures and images for: TGF-β1 conjugated chitosan collagen hydrogels induce chondrogenic differentiation of human synovium-derived stem cells
Source: J Biol Eng. 2015 Jan 14;9:1. doi: 10.1186/1754-1611-9-1 (PMC4350967; doi:10.1186/1754-1611-9-1)

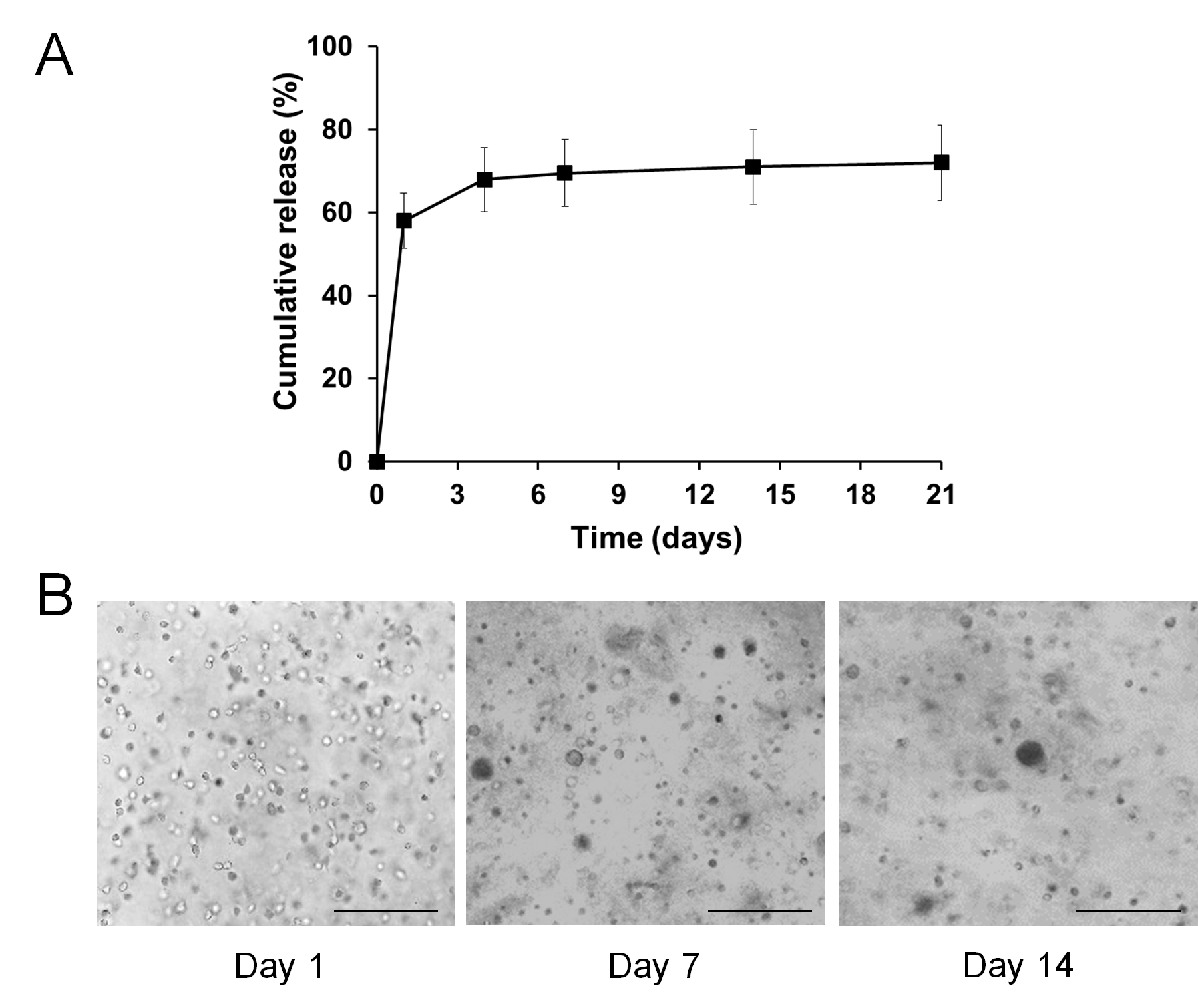

Supplement: Supplementary file 1 — Additional file 1: Figure S1: Supplementary data of TGF-β1 release (A) and SMSC growth (B) in MeGC/Col containing non-specifically adsorbed TGF-β1. Scale bar = 200 μm. (TIFF 10 MB) [file 13036_2014_160_MOESM1_ESM.tiff]

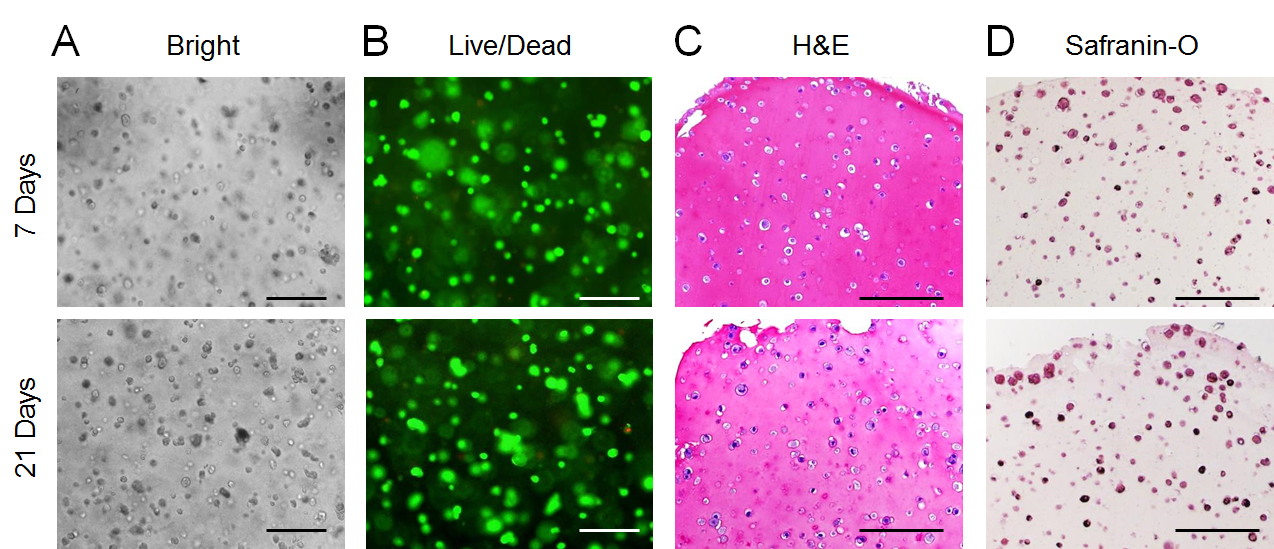

Supplement: Supplementary file 2 — Additional file 2: Figure S2: Supplementary data of SMSC cultured in MeGC/TGF without collagen at day 21 including (A) Bright field images, (B) live/dead staining, (C) H & E staining, and (D) Safranin-O staining. Scale bar = 100 μm. (TIFF 4 MB) [file 13036_2014_160_MOESM2_ESM.tiff]
